# The Animal Lectin Galectin-8 Promotes Cytokine Expression and Metastatic Tumor Growth in Mice

**DOI:** 10.1038/s41598-020-64371-z

**Published:** 2020-04-30

**Authors:** Hadas Shatz-Azoulay, Yaron Vinik, Roi Isaac, Ulrike Kohler, Sima Lev, Yehiel Zick

**Affiliations:** Department of Molecular Cell Biology, Weizmann Institute of Science, Rehovot, 7610001 Israel

**Keywords:** Metastasis, Glycobiology

## Abstract

Secreted animal lectins of the galectin family are key players in cancer growth and metastasis. Here we show that galectin-8 (gal-8) induces the expression and secretion of cytokines and chemokines such as SDF-1 and MCP-1 in a number of cell types. This involves gal-8 binding to a uPAR/LRP1/integrin complex that activates JNK and the NFkB pathway. Cytokine and chemokine secretion, induced by gal-8, promotes migration of cancer cells toward cells treated with this lectin. Indeed, immune-competent gal-8 knockout (KO) mice express systemic lower levels of cytokines and chemokines while the opposite is true for gal-8 transgenic animals. Accordingly, gal-8 KO mice experience reduced tumor size and smaller and fewer metastatic lesions when injected with cancer cells. These results suggest the existence of a ‘vicious cycle’ whereby gal-8 secreted by the tumor microenvironment, promotes secretion of chemoattractants at the metastatic niche that promote further recruitment of tumor cells to that site. This study further implicate gal-8 in control of cancer progression and metastasis through its effects on the production of immunoregulatory cytokines.

## Introduction

Breast and prostate cancer are major contributors to cancer-related death in the Western world^1–3^ with lungs and bones being preferred metastatic sites. Bone metastasis results in osteoblastic lesions that progress to osteolytic lesions in late stages of the disease^3,4^. It involves interactions between cancer cells and the bone tissue with active participation of osteoblasts^5,6^, in a manner yet incompletely understood^4–6^.

Animal lectins of the galectin family emerge as key players in the process of cancer growth and metastasis^7,8^. Galectins are devoid of signal sequences and are secreted by an atypical secretory pathway. As extracellular ligands they activate a number of signaling pathways that result in cell adhesion and cytoskeletal remodeling, immune response, inflammation, cell proliferation, and apoptosis [cf.^9,10^ for recent reviews]. Galectins regulate tumor progression and metastasis by affecting the interactions between tumor, stromal, immune, and endothelial cells^11^. Yet, in spite of the wealth of information concerning their expression in cancerous tissues^7,12^, the functional role of galectins in cancer progression and metastasis remains incompletely understood.

Galectin-8 (gal-8)^13^ is a tandem-repeat type galectin, having two carbohydrate-recognition domains (CRDs) joined by a linker peptide^13,14^. Gal-8, like other galectins, is secreted by a number of cell types^15,16^ and is present at high concentrations extracellularly (e.g. synovial fluids of RA patients^17^ or serum of cancer patients^18^). Secreted gal-8 functions as an extracellular matrix protein when immobilized, and induces cell adhesion upon binding to CD44 and a subset of integrins^17,19–21^. Gal-8 and integrins form complexes that involve sugar-protein interactions. This activates signaling cascades including phosphorylation of paxillin and focal adhesion kinase (FAK), followed by persistent activation of PI3K and the Extracellular signal-regulated kinase (ERK)^19,20,22,23 ^.

Selective amplification of the gal-8 gene (LGALS8) and increased gal-8 expression is observed in a number of cancerous tissues^12,24–26^,  including breast, prostate, and lung^12,24,25,27,28^ and is often associated with poor prognosis^12^. For example, gal-8 concentration is elevated in sera of colon and breast cancer patients, where it supports adhesion of tumor cells to the microvascular lung endothelium^29^. Similarly, gal-8 promotes adhesive interactions between multiple myeloma and vascular endothelial cells^30^, while binding of lung cancer cells to a complex of fibronectin and gal-8 results in metastatic growth of lung adenocarcinoma^31^. Conversely, the frequency of lymph node metastasis is completely abolished upon silencing of gal-8 in a mouse model of prostate cancer^28^. Still, a number of studies reported on decreased expression of gal-8 in association with favorable early tumor progression^32^, suggesting that its mode of action in cancer growth and metastasis is more complex than initially envisaged.

Tumor invasiveness and metastatic dissemination are also regulated by immunomodulators^33^ that serve as maintenance and survival factors of cancer cells^6^. The role of galectins in controlling immune regulatory cancer networks has been studied to a certain extent^7^ however the function of gal-8 in these processes remains elusive^18^. To address this issue, we made use of primary osteoblast cultures and mice overexpressing gal-8, to show that gal-8 significantly increases in target tissues the production and secretion of cytokines and chemokines that chemoattract cancer cells. Conversely, tumor growth and metastasis were markedly diminished in immune-competent gal-8 knockout mice that manifest a systemic reduction in cytokines/chemokines expression. These findings implicate gal-8 as a potential promoter of immune-regulatory networks that induce chemoattraction and cancer progression at the metastatic niche.

## Results

### Gal-8 induces expression of pro inflammatory cytokines and metastasis-related chemokines

**Figure 1:**
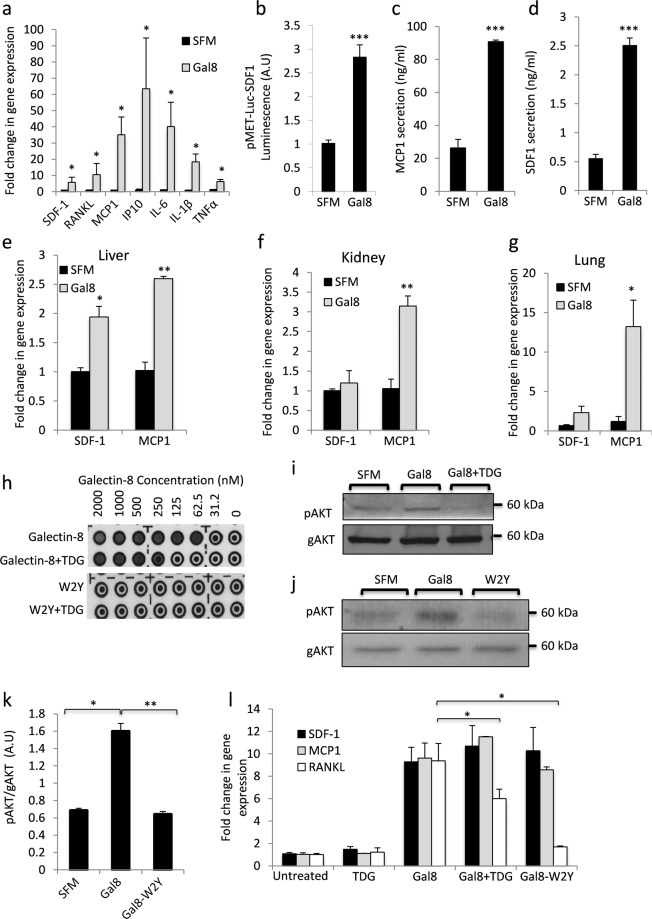
Gal-8 induces expression of cytokines and chemokines in different organs. (**a**) Osteoblasts extracted from calvariae of newborn CD1 mice were incubated with or without 50 nM gal-8 for 24 h. Cells were harvested, total mRNA was extracted and qRT-PCR was conducted. HPRT served as a control for normalization purposes. Results shown are means ± SEM of 7–11 experiments done in duplicates. (**b**) Osteoblasts were transfected with pMET-Luc-SDF-1 vector. 24 h post transfection the cells were incubated in the presence or absence of 50nM gal-8 for 24 h, and luciferase levels in the conditioned medium were determined. Media from the above cells were used (**c, d**) to quantify the amounts of secreted SDF-1 (**d**) and MCP-1 (**c**) proteins. Results shown are means ± SEM of 3 experiments done in 5–8 repeats (**b**) or of two experiments done in triplicates (**c**,**d**). Liver (**e**), kidneys (**f**) and lungs (**g**) were removed from nine-week old CD1 mice. Single cell suspensions were made and were treated with 50nM gal-8 or serum-free medium (SFM; control) for 24 h. Cells were harvested, total mRNA was extracted and qRT-PCR was conducted for SDF-1 and MCP-1. HPRT served as a control for normalization purposes. Results shown (e-g) are means ± SEM of 3 experiments done in duplicates. (**h**) The ability of 20 mM TDG to inhibit the hemagglutination activity of the indicated concentrations of gal-8 or its GST-W2Y gal-8 mutant were determined. Results shown are of a representative experiment repeated at least 6 times. (**i**,**j**) Primary osteoblasts were treated with GST-gal-8 in the absence or presence of 20 mM TDG (**i**), or with GST-W2YGal8 (50 nM) for 30 min (**j**). Cells were harvested, total proteins were extracted and were analyzed by Western blotting using antibodies specific for the phosphorylated forms of AKT (pAKT) or total general AKT (gAKT). A representative experiment is shown in (**i**,**j**) and quantification of three similar experiments is shown in (**k**). (**l**) Primary osteoblasts were treated for 24 h with gal-8 (50 nM) or GST-W2YGal-8 (50 nM), with or without TDG (20 mM). Cells were harvested, total mRNA was extracted and qRT-PCR was conducted to quantify the mRNA levels of SDF-1, MCP-1 and RANKL. Actin mRNA served as a control for normalization purposes. Results are mean ± SEM of 3 experiments done in duplicates (*p < 0.05; **p < 0.01; ***p < 0.001 vs. untreated controls). The full-length blots of (**i**,**j**) are shown in Supplemental Fig S10.

Galectin-8 is a secreted animal lectin, expressed by various cell types including primary osteoblasts (cf. ref. ^15,16^ and Supplemental Fig. S1). We have previously shown that gal-8 promotes the expression of the cytokine Receptor Activator of NFkB ligand (RANKL) in primary osteoblasts^16^. Given that tumor growth and metastasis are regulated by chemokines and pro-inflammatory cytokines^33^ and since bone is a preferred metastatic niche, it was of interest to determine whether gal-8 affects cytokine and chemokine expression in bones. Recombinant gal-8 and primary osteoblasts extracted from calvariae of CD1 newborn mice were used for that purpose. Treatment of osteoblasts with gal-8 for 24 h significantly increased 5–60 fold the mRNA levels of a number of chemokines and cytokines including SDF-1, Tumor necrosis factor (TNF)-α, RANKL, interleukin -1 (IL-1)β, MCP-1, IP10 and IL-6 (Fig. 1a). Microarray analysis of RNA derived from naïve osteoblasts treated with gal-8 revealed increased expression of additional chemokines (e.g. Ccl9, Cxcl10, Ccl7) (Table 1), suggesting that gal-8 is a general regulator of chemokine expression.

**Table 1:** Osteoblasts extracted from calvariae of 1-d old mice and were treated with 50 nM gal-8 for 24 h in serum-free medium (SFM). RNA was extracted and taken for microarray analysis. Shown are changes in chemokines expression between gal-8-treated and non-treated osteoblasts.

^a^Gene Symbol	p-value	Fold change (Gal8 vs. Control)
Cxcl16	7.10E-04	2.3
Ccl9	9.01E-04	2.5
Ccl11	1.12E-04	4.4
Cxcl12	3.40E-04	5.0
Ccl7	1.56E-04	9.6
Ccl3	8.13E-05	11.5
Cxcl10	4.16E-03	11.9
Cxcl5	1.43E-05	12.9
Ccl2	8.12E-05	18.09
Cxcl9	1.64E-03	21.79
Cxcl1	8.27E-07	24.7
Ccl5	3.29E-04	37.0

^a^NCBI Reference Sequence Database.

Given that SDF-1 and MCP-1 were implicated in cancer metastasis to bone^34^, the effects of gal-8 on their expression were further investigated. Using p-Met-Luciferase vector, cloned downstream of the SDF-1 promoter, we could show that osteoblasts transfected with this construct and treated with gal-8 exhibited significantly higher (2.5 fold) luciferase activity than osteoblasts treated with serum-free medium (SFM) (control) (Fig. 1b). We could further show that naïve osteoblasts treated with gal-8 manifested a 5 fold increase in MCP-1 and SDF-1 secretion (Fig. 1c,d). The effects of gal-8 were not limited to primary osteoblasts. Gal-8 increased the mRNA levels of MCP-1 by 2.5, 3 and 10 fold in liver,  kidney and lung, respectively while SDF-1 expression increased 2–3 fold in cells isolated from liver and lung (Fig. 1e–g). These results suggest that the stimulatory effects of gal-8 on SDF-1 and MCP-1 expression might be a general phenomenon observed in a number of cell types and could have a physiological effect in promoting metastasis through the induction of SDF-1 and MCP-1 expression and secretion.

### Gal-8 induction of SDF-1 expression is independent of its sugar binding properties

Gal-8 acts as an extracellular ligand that activates signaling pathways both by protein–sugar and protein–protein interactions^14^. To determine how gal-8 triggers SDF-1/MCP-1 transcription, its sugar-binding activity was blocked by thiodigalactoside (TDG)^14^. Alternatively, we used a GST-gal-8 mutant, denoted W2Y, that lacks sugar-binding activity, due to mutation of two Trp residues (W85 and W248) crucial for sugar binding^14^ into Tyr. Indeed, we could show that 20 mM TDG effectively inhibited the hemagglutination activity of gal-8, while the W2Y gal-8 mutant showed no such activity (Fig. 1h). We could further demonstrate (Fig. 1i) that TDG inhibited gal-8-mediated Akt (Protein kinase B (PKB)) phosphorylation, one of the hallmarks of gal-8’s activity that involves protein-sugar interactions^23^. Similarly, unlike the naive gal-8, its W2Y mutant failed to stimulate Akt phosphorylation (Fig. 1j,k; Supplemental Fig. S2-a). These results indicated that indeed the W2Y gal-8 mutant lacks bioactivity which depends upon sugar binding. Next, the effects of TDG and those of GST-gal-8-W2Y on cytokine expression were evaluated. As shown in Fig. 1l, TDG partially inhibited RANKL expression induced by gal-8, while having no inhibitory effects on SDF-1/MCP-1 expression. Accordingly, the gal-8-W2Y mutant failed to induce RANKL expression while it was equipotent to naive gal-8 in promoting SDF-1/MCP-1 expression. These results indicate that gal-8’s effects on SDF-1 and MCP-1 in osteoblast are independent of its sugar binding properties, whereas its effects on RANKL expression involve, at least in part, its sugar binding ability.

### Gal-8 promotes chemoattraction of prostate cancer cells toward osteoblasts via SDF-1 and MCP-1

**Figure 2:**
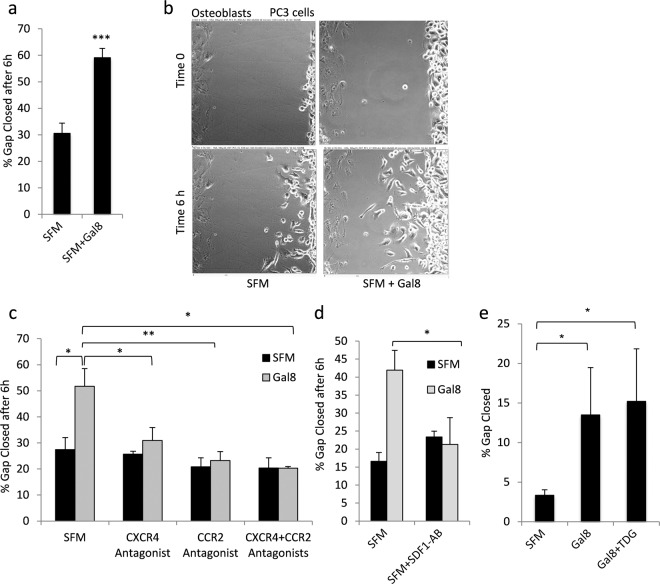
SDF-1 and MCP-1, secreted by gal-8-treated osteoblasts, chemoattract cancer cells. (**a**–**e**) Osteoblasts (70,000 cells) were seeded in one chamber of ‘ibidi’ Culture-Inserts and were incubated at 37 ^o^C. 24 h later the osteoblasts were treated with gal-8 (50 nM), SFM (control) (**a**–**d**), or gal-8+TDG (20 mM) (**e**) while PC3 cells (35,000 cells) were seeded in the second chamber. 24 h thereafter the culture-media were replaced with gal-8-free medium. The ‘ibidi’ inserts were removed and the cells were further incubated at 37 °C for 6 h. The gap between the two cultures was photographed and quantified at the time of removal of the ‘ibidi’ insert (time 0) and 6 h later. n = 10 independent experiments carried out in duplicates (**a**). A representative picture is shown in (**b**). CXCR4 antagonist AMD3100 (90 μM); CCR2 antagonist (6 nM) (**c**); SDF-1 Antibodies (2 μg/ml) (**d**) were added to the culture medium at the time the inserts were removed and cell migration was assayed at time 0, and 6 h. Results shown are means ± SEM of 4 (**a**–**c**) or 3 (**d**,**e**) experiments done in duplicates (*p < 0.05; **p < 0.01; *** p< 0.001 vs. untreated controls).

Given that gal-8 induces expression and secretion of chemokines in different organs, we wished to determine whether it affects the migration of prostate cancer cell towards osteoblasts. Osteoblasts were seeded in one chamber of ‘ibidi’ culture-insert and were treated with or without gal-8 (50 nM, 24 h). Human prostate cancer (PC3) cells^35^ were then seeded in the next ‘ibidi’ chamber. The ‘ibidi’ inserts were removed 24 h later and the PC3 cells were allowed to migrate for 6 h and close the gap between them and the osteoblasts. Treatment of osteoblasts with gal-8, prior to their interaction with the PC3 cells, increased ~2 fold PC3 cells migration towards these osteoblasts (Fig. 2a). Treatment of PC3 cells themselves with gal-8 did not affect their migration towards non-treated PC3 cells, while the osteoblasts themselves also failed to significantly migrate within the 6 h time frame of the experiments (Supplemental Fig. S3). These results suggest that gal-8 promotes chemoattraction of PC3 cells towards osteoblasts. Treatment of PC3 cells with Actinomycin-D that inhibits cell proliferation did not affect their accelerated migration towards gal-8-treated osteoblasts (Supplemental Fig. S4), indicating that the gap closure by PC3 cells is due to migration rather than extensive proliferation. Finally, PC3 cell migration towards gal-8-treated osteoblasts was recorded using live-cell imaging as described^36^. As shown in a snap-shot captured 6 h after initiation of the experiment (Fig. 2b), we observed accelerated migration of PC3 cells towards gal-8-treated osteoblasts, rather than PC3 cell proliferation.

The enhanced migration of PC3 cells (Fig. 2b) was indeed mediated by SDF-1 and MCP-1, secreted by gal-8-treated osteoblasts, because AMD3100, the SDF-1 receptor (CXCR4) inhibitor^37^, or the MCP-1 receptor (CCR2)-antagonist^38^, either alone or in combination, effectively abolished the stimulatory effects of gal-8 on PC3 cell migration toward osteoblasts (Fig. 2c). Inclusion of specific SDF-1 antibodies but not control IgG, (Supplemental Fig S5) that prevents SDF-1 interactions with CXCR4 also reduced PC3 cells migration toward gal-8-treated osteoblasts (Fig. 2d). Gal-8-induced gap-closure, like its effects on cytokine secretion (Fig. 1l) were sugar-independent as inclusion of TDG failed to inhibit this process (Fig. 2e). These results conform with our hypothesis that gal-8 induces SDF-1 and MCP-1 secretion from osteoblasts and that those chemokines facilitate prostate cancer cell migration towards their target tissues. Of note, we observed certain variance in the percentage of gap closure in different experiments (e.g. Fig. 2c vs. 2e). This could be attributed to the fact that different batches of freshly isolated primary osteoblasts were used in the different experimental sets.

### Gal-8 induction of SDF-1 and MCP-1 involves its binding to the low density lipoprotein receptor-related protein (LRP)-1 and the urokinase plasminogen activator receptor (uPAR) and activation of the c-Jun N-terminal kinase (JNK) and Nuclear factor kappa light-chain-enhancer of activated B cells (NFκB) signaling pathways

**Figure 3:**
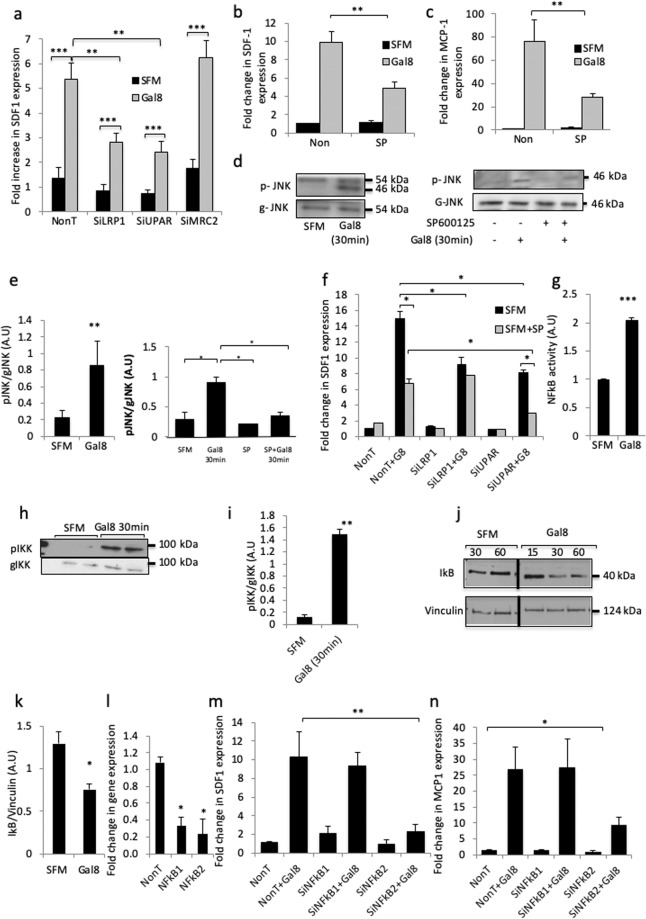
The LRP1 and uPAR receptors and the JNK and NFkB signaling pathways mediate the effects of gal-8 on SDF-1 and MCP-1 expression in primary osteoblasts. (**a**) Osteoblasts (5 × 10^4^ cells) were transfected with siRNAs directed against LRP1, uPAR, MRC2 or control non-targeting (NonT) siRNA. 72 h thereafter gal-8 (50 nM) was added for another 24 h, after which the cells were harvested, total mRNA was extracted and qRT-PCR was conducted for SDF-1 mRNA. HPRT served as a control for normalization purposes. (**b**–**e**) Osteoblasts were treated with SP600125 (10 µM). 2 h thereafter gal-8 (50 nM) was added for 24 h (**b**,**c**) or 30 min (**d**,**e**). Cells were harvested, total mRNA was extracted and qRT-PCR was conducted to quantify the mRNA levels of for SDF-1 (**b**) and MCP-1 (**c**). HPRT served as a control for normalization purposes. Results shown are means ± SEM of 4 experiments done in duplicates. (**d**, **e**) Osteoblasts (10^5^ cells) were treated with SP600125 (10 µM). 2 h thereafter gal-8 (50 nM) was added for additional 30 min. Total proteins were extracted and analyzed by Western blotting using p-JNK specific antibodies. Anti-general JNK (g-JNK) (**d**) served as control. Data shown in (**e**) summarizes 2 experiments quantified by densitometry. (**f**) Osteoblasts (5 × 10^4^ cells) were transfected with LRP1 or uPAR siRNAs (or control non-targeting siRNA). After 48 h SP600125 was added for 2 h and then gal-8 (50 nM) was added for additional 24 h, after which the cells were harvested, total mRNA was extracted and qRT-PCR was conducted in order to quantify changes in SDF-1 mRNA. HPRT served as a control. Results shown are means ± SEM of 3–4 experiments done in duplicates. (**g**) Osteoblasts (3 × 10^3^ cells) were transfected with p-MET-LUC-NFkB construct. 24 h post transfection, cells were treated with gal-8 (50 nM) for 4 h. Conditioned medium was collected and luciferase secretion was determined. (**h**–**k**) Osteoblasts (10^5^ cells) were treated with gal-8 (50 nM) for 30 min (**h**,**i**,**k**) or for the indicated time periods **(j**). At the end of incubation total proteins were extracted and analyzed by Western blotting using antibodies specific for the phosphorylated forms of IKKα/β (**h**,**i**) or IkB (**j**,**k**). Anti-general IKK (g-IKK) (**h**) or vinculin (**j**) served as controls. Results of 3 experiments done in duplicates were quantified (**i**,**k**). (**l**–**n**) Osteoblasts (5×10^4^ cells) were transfected with NFkB1 (p105), NFkB2 (p100) siRNAs, or with control non-targeting siRNA. 48 h thereafter gal-8 (50 nM) was added for additional 24 h, after which the cells were harvested, total mRNA was extracted and qRT-PCR was conducted in order to quantify changes in mRNA levels of NFkB1 and NFkB2 (**l**) SDF-1 (**m**) or MCP-1 (**n**). Actin mRNA served as a control for normalization purposes. Results shown are means ± SEM of 3 experiments done in duplicates. (*p < 0.05; **p < 0.01; ***p < 0.001 vs. untreated controls). The full-length blots of (**d,h,j**) are presented as Supplemental Figs. S11–S13, respectively. Vertical black lines (**j**) represent positions where the original blots were cropped and non-relevant lanes were omitted.

We have previously shown that binding of gal-8 to MRC2/LRP1/uPAR receptor complexes in osteoblasts induces RANKL expression by these cells^16^. Therefore, we investigated the role of these receptors in mediating SDF-1 and MCP-1 secretion from osteoblasts treated with gal-8. Osteoblasts were transfected with small interfering RNAs (siRNAs) against LRP1, MRC2 or uPAR that silenced their expression by 80–90% each^16^ (cf. also Supplemental Fig. S6). Next, the cells were treated with gal-8 (50 nM, 24 h). Silencing of uPAR and LRP1 resulted in ~50% decrease in SDF-1 transcription in response to gal-8, compared to cells treated with control siRNA. However, silencing of MRC2 did not impede the stimulatory effects of gal-8 on SDF-1 expression (Fig. 3a). These results suggest that the effects of gal-8 on SDF-1 expression in osteoblasts involve its binding to LRP1 and uPAR.κ

To identify the signaling pathways mediating the effects of gal-8, primary osteoblasts were treated with MEK, Akt and p38 inhibitors, but these did not affect its ability to induce SDF-1/MCP-1 expression (Supplemental Fig. S7). In contrast, the JNK inhibitor SP600125 reduced SDF-1 and MCP-1 expression in response to gal-8 (Fig. 3b,c). Accordingly, and consistent with our previous studies in other cell types^23^, gal-8 induced phosphorylation (activation) of JNK in primary osteoblasts, and SP600125 inhibited this effect (Fig. 3d,e). These results suggest that JNK mediates the effects of gal-8, but additional signaling pathways are involved as well.

To explore the possible role of JNK as a downstream effector of LRP1 and uPAR, osteoblasts were transfected with LRP1 and uPAR siRNAs. 48 h post transfection the cells were further treated with SP600125 to inhibit JNK phosphorylation and then gal-8 was added for 24 h. We could show (Fig. 3f) that inhibition of JNK combined with silencing of uPAR had significant additive inhibitory effects on SDF1 transcription in response to gal-8, suggesting that uPAR signaling induced by gal-8 is not mediated by JNK. In contrast, the inhibitory effects due to silencing of LRP1 and JNK were not additive (Fig. 3f), suggesting that JNK is activated upon gal-8/LRP1 interactions.

Since MCP-1 and SDF-1 transcription is regulated by the (NFkB)^39,40^ we analyzed the effects of gal-8 on this pathway in osteoblasts. Indeed, we could show a 2 fold increase in NFkB transcription activity in osteoblasts transfected with a pNFkB-MetLuc2-reporter that were treated with gal-8 for 4 h (Fig. 3g). This was accompanied by 3–4 fold increased phosphorylation (activation) of IKKα/β, the upstream activator of NFkB that was already evident after 3 min treatment of osteoblasts with gal-8 (Supplemental Fig. S8) and persisted for at least 30 min (Fig. 3h,i). A corresponding 40% reduction in protein levels of IkB, the downstream target of IKKβ, and the upstream activator of the NFkB pathway, was detected 30 min following treatment with gal-8 (Fig. 3j,k).

To establish NFkB as a mediator of gal-8’s effects on SDF-1 and MCP-1 transcription, we silenced p100 and p105, the precursors of NFkB1 and NFkB2, respectively^41^. As shown in Fig. 3l the mRNA levels of p105 and p100 were reduced by 70–80% by their respective siRNAs. Furthermore, silencing of NFkB2 decreased the expression of SDF-1 and MCP-1, induced by gal-8, by 75%, and 65%, respectively, while silencing of NFκB1 had no such an effect (Fig. 3m,n). These results suggest that NFκB2 mediates, at least partially, SDF-1 and MCP-1 transcription in response to gal-8.

### Alterations in cytokine/chemokine secretion in gal-8-transgenic (Tg) and knockout (KO)-mice

**Figure 4:**
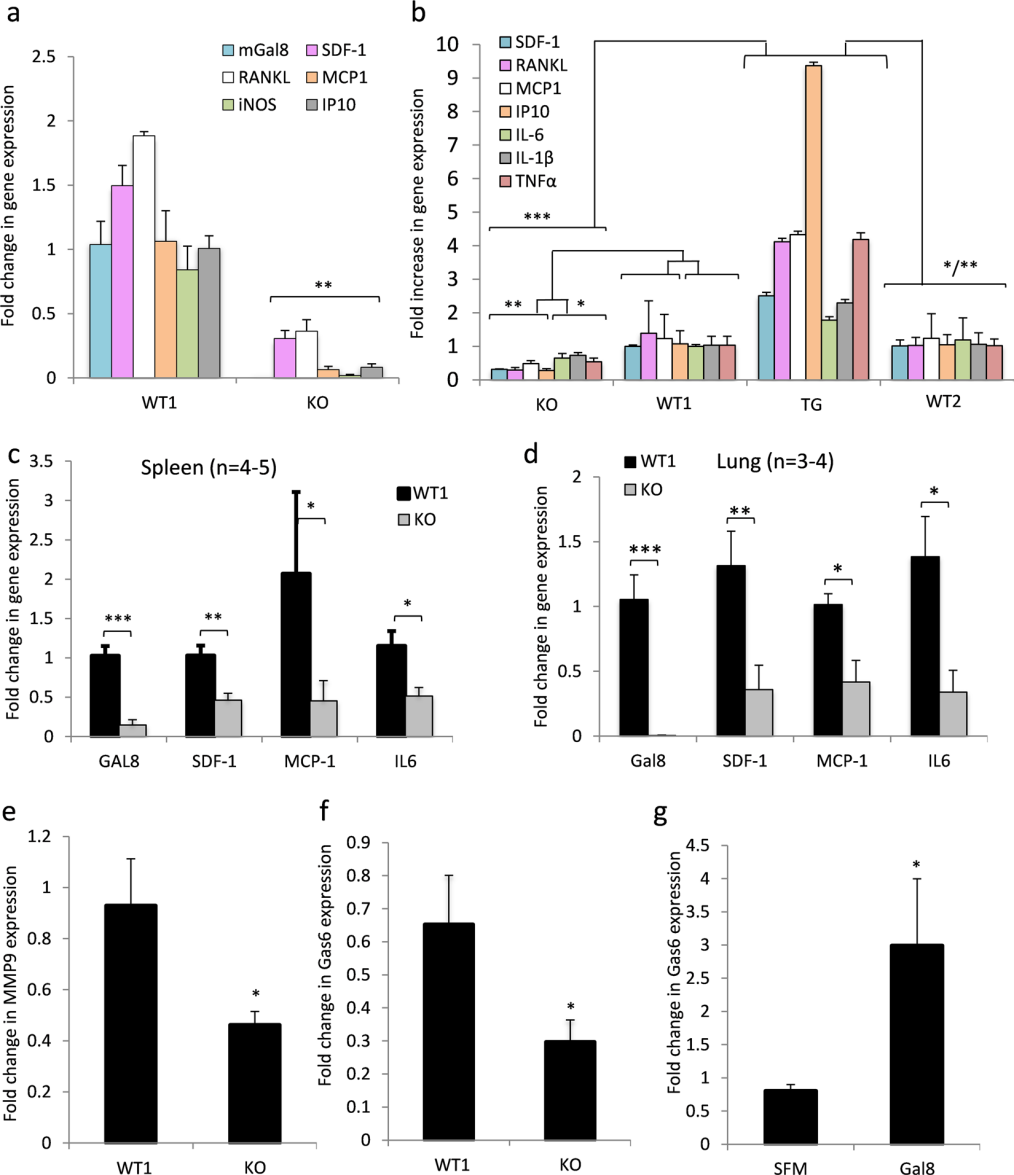
mRNA levels of cytokines and chemokines in osteoblasts derived from gal-8 KO and gal-8 Tg mice. (**a**) Osteoblasts derived from calvariae of newborn gal-8 KO mice and their WT control littermates (*WT1*) were seeded in 6-well plate (10^5^ cells per well) and incubate at 37 ^o^C for 24 h. Cells were then harvested; total mRNA was extracted and qRT-PCR was conducted to quantify the indicated mRNA levels. Results shown are means ± SEM of 4 experiments done in duplicates. (**b**) mRNA was extracted from osteoblast derived from long bones of 14–15 weeks old gal-8 KO and Tg mice and their WT controls (*WT1 and WT2, respectively*). qRT-PCR was conducted to quantify the indicated mRNA levels. Results shown are means ± SEM of 4 experiments done in duplicates. (**c**,**d**) mRNA was extracted from spleen (**c**) and lung (**d**) derived from 7-week-old *WT1* and gal-8 KO mice. qRT-PCR was conducted to quantify the indicated mRNA levels. (**e**,**f**) mRNA was extracted from long bones of adult WT1 and gal-8 KO. qRT-PCR was conducted to quantify the mRNA levels of MMP9 (**e**) and Gas-6 (**f**) (n = 4–6 mice/group). (**g**) Osteoblasts (1 × 10^5^ cells/well) extracted from calvariae of newborn CD1 mice were treated with 50 nM gal-8 for 24 h. Cells were harvested, total mRNA was extracted and qRT-PCR was conducted to determinate Gas-6 mRNA levels. Actin served as a control for normalization purposes. Results shown are means ± SEM of 4 experiments done in triplicates. [*p < 0.05; **p < 0.01; ***p < 0.001 vs. WT mice (**a**–**f**) or untreated controls (**g**)].

To determine the physiological significance of the above findings we studied the levels of expression of cytokines/chemokines in gal-8 Tg and gal-8 KO mice. As expected, no expression of gal-8 mRNA itself was detected in osteoblasts isolated from newborn gal-8 KO mice (Fig. 4a and^42^). Furthermore, the mRNA levels of a number of cytokines and chemokines including MCP-1 and SDF-1 were significantly reduced (by 80–95%) in osteoblasts derived from these animals, when compared to wild-type (WT) mice (Fig. 4a). We also observed a 95% decrease in mRNA levels of inducible nitric oxide synthase (iNOS) that can be activated by pro-inflammatory cytokines.

Gal-8 Tg mice presented a mirror image to that of gal-8 KO mice. The mRNA levels of a number of cytokines (i.e. MCP-1, SDF-1, IP-10, IL-6, IL-1β, TNF-α), in addition to RANKL^16^, were increased in long bones of 14–15 weeks old mice, when compared to WT mice (Fig. 4b), while the opposite was true for gal-8 KO mice. These results establish the role of gal-8 as a physiological regulator of cytokine/chemokine expression. To determine whether the reduced expression of cytokines/chemokines in gal-8 KO mice is indeed a systemic effect, mRNA was extracted from lungs and spleens of 7-weeks old mice. As expected, gal-8 KO mice did not express gal-8 mRNA in these tissues while the mRNA levels of IL-6, SDF-1, and MCP-1 were decreased 2–4 fold when compared to their WT controls (Fig. 4c,d). These results further establish gal-8 as a physiological systemic regulator of cytokine and chemokine expression in different tissues and cell types.

### Gal-8 KO mice express lower levels of MMP9 and Gas6

Cytokines such as SDF-1 up regulate gene expression of MMPs^43^ that play key roles in promoting cancer metastasis^44,45^. Therefore, we aimed to determine whether the mRNA levels of MMP9 are altered in gal-8 KO mice. Using RNA extracted from long bones of Gal8-KO mice we found significantly lower (50%) mRNA levels of MMP9 in gal-8 KO mice when compared to WT mice (Fig. 4e), suggesting that this might also contribute to the resistance of Gal-8 KO mice to develop cancer metastasis.

Growth arrest-specific gene 6 (Gas6), the ligand of the TAM family (Tyro3, Axl, and Mer) of receptor tyrosine kinases, is another downstream target of SDF-1^46^. Gas6 is frequently expressed in cancers and its levels correlate with poor prognosis^47^. Indeed, Gas6 expression was significantly reduced (~50%) in osteoblasts derived from Gal-8 KO mice (Fig. 4f). Accordingly, gal-8 could significantly stimulate (~4–6 fold) Gas6 expression in primary cultured osteoblasts treated with this lectin (Fig. 4g), thus providing a direct physiological link between gal-8 and Gas6 expression.

### Gal-8 promotes cancer growth and metastasis *in-vivo*

**Figure 5:**
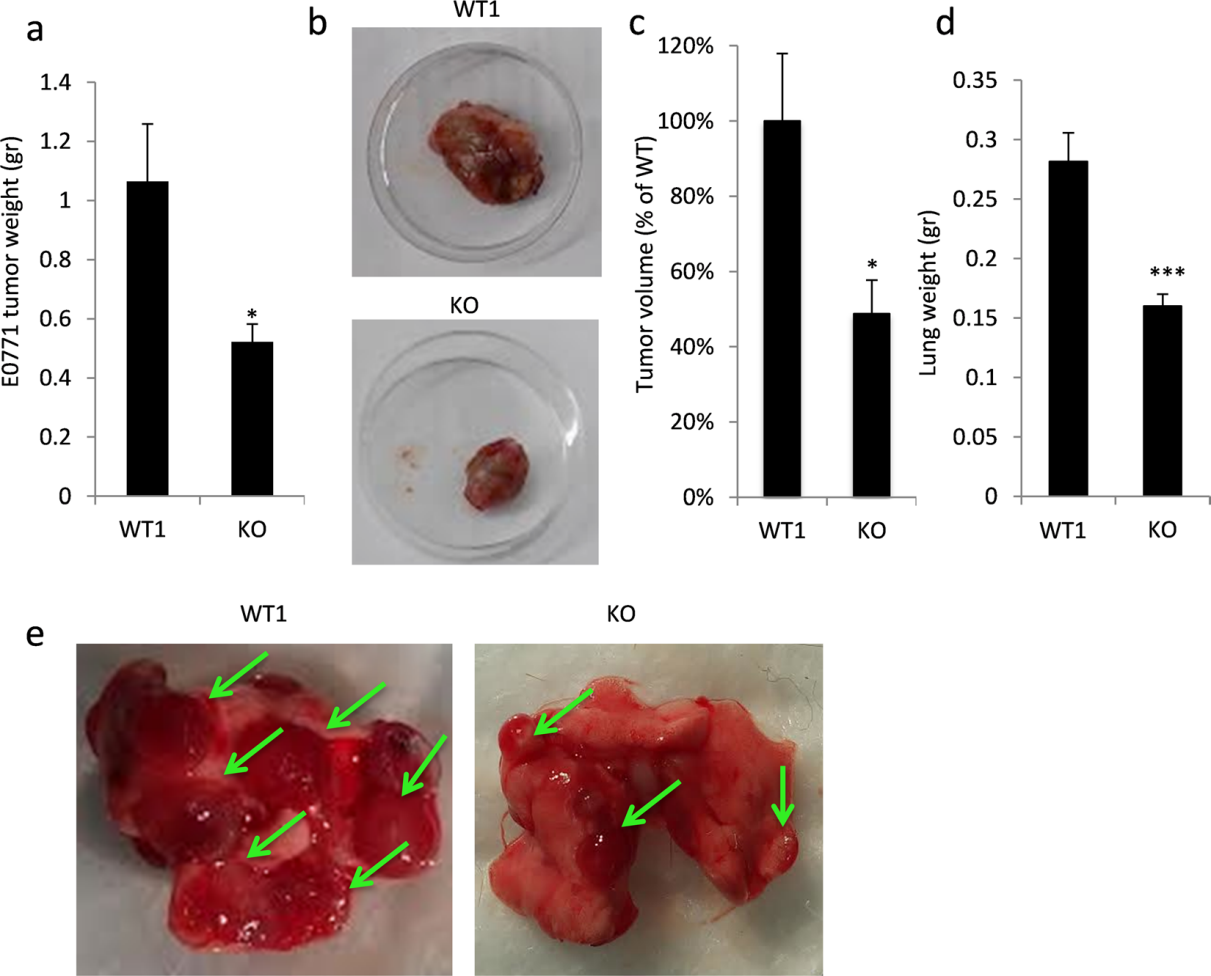
Gal-8 KO mice manifest reduced growth and metastatic potential of cancer cells *in vivo*. (**a**–**c**) 5 × 10^5^ E0771 cells were injected orthotopically into the 4^th^ mammary gland of WT1 or gal-8 KO female mice (9 weeks old). 3 weeks post injection the mice were sacrificed and the tumor developed in their mammary glands were weighted (**a**) n = 5 mice/group. A representative photograph is shown in (**b**). Changes in tumor volume are shown in **c**. (**d**,**e**) 5 × 10^5^ D122-Luc cells were injected into the tail vein of WT1 or gal-8 KO male mice (9 weeks old). 6 weeks post injection mice were sacrificed and their lungs were extracted. Lungs weight (**d**) were examined (n = 6). (*p < 0.05; *** p < 0.001 vs. WT control littermates). A representative image of tumor nodules in the lungs is shown in (**e**).

Given that cytokines and chemokines play key roles in tumor progression *in vivo*^33^ and given that gal-8 promotes cytokine and chemokine expression in mice, we studied the effects of its depletion on cancer growth and metastasis. The proper model to study should have been the bone metastatic niche, however such a model is difficult to establish in immune competent C57Bl/6 J mice. Therefore, other models were employed. E0771 breast cancer cells^48^ and D122-Luc Lewis lung carcinoma cells^49^, derived from C57BL6/J mice, that share isogeneic background with gal-8 KO mice were used. As shown in Fig. 5a–c (and Supplemental Fig. S9a,b) orthotopic injection of E0771 cells to the 4^th^ mammary gland of gal-8 KO female mice resulted in development of significantly lighter (~40%) primary tumors, having smaller size and volume than tumors that grew in WT mice. Similarly, injection of D122-Luc cells to the tail vein of gal-8 KO male mice resulted in development of lung metastatic lesion, having reduced weight (Fig. 5d) and fewer lesions (Fig. 5e) when compared to metastatic lesions developed in their WT control littermates (cf. also Supplemental Fig. S9c). These results suggest that the lower levels of cytokines/chemokines expressed in gal-8 KO mice may contribute to the reduced formation of primary tumors and metastatic lesions in these animals.

## Discussion

The present study employes cultured cells and immune competent mouse models to suggest the existence of a ‘vicious cycle’ whereby gal-8, secreted by tumor cells and their microenvironment, induces cytokine and chemokine production that supports further growth of primary tumors and metastatic lesions. Expression of additional mediators of tumor growth, including MMP9 and Gas6 is also induced by gal-8. The underlying mechanism involves binding of gal-8 to a complex of cell surface receptors that include LRP1 and uPAR; activation of the JNK and NFkB signaling pathways and induction of cytokine/chemokine production (e.g. MCP-1 and SDF-1). It occurs in a cell-autonomous manner in a number of cell types and tissues, suggesting that gal-8 induces a systemic response *in vivo*. This mechanism might be of clinical relevance, as the levels of expression of gal-8 at least in human bones, positively correlate with those of RANKL^16^ and SDF-1 *(unpublished*).

Cytokines and chemokines are well known chemo-attractants that stimulate migration of malignant cells towards their metastatic niche^33^. Chemokine receptors are expressed by different cancer cells^50^ and up-regulation of chemokine-receptor pairs (e.g. [Stromal cell-derived factor 1 (SDF-1)/ C-X-C chemokine receptor type 4 (CXCR4)] promotes metastasis^33^. CXCR4 enhances metastatic growth of breast cancer cells to bone, liver, and lung, tissues in which its ligand SDF-1 is expressed in high amounts^51,52^. SDF-1/CXCR4 signaling also benefits cancer cells by elevating the cells ability to express and secrete matrix metalloproteinases (MMPs) such as MMP9^53^. Similarly, Monocyte chemoattractant protein- 1 (MCP-1) also known as CCL2 (C-C motif chemokine ligand 2), and its receptor CCR2 (C-C chemokine receptor type 2) were linked to tumor growth and poor prognosis in colon, breast, prostate, and cervical cancer^54^.

Certain effects of galectins^7,12^, including gal-8, on immune regulatory cancer networks were explored. For example, HMEC-1 cells treated with gal-8 produce CXCL8 (IL-8), CXCL3 (GRO-γ), CCL5 (RANTES), CXCL1 (GRO-α), Macrophage colony stimulating factor (M-CSF), IL-6, and CCL2 (MCP-1) in a process that requires activation of NFkB^55^. Gal-8 also increases secretion of chemokines and cytokines (i.e. IL-2, MCP-5, IL-6, MCP-1, TNF-α,, and IL-3) from bone marrow-derived dendritic cells^56^. Most relevant are the observations that gal-8 present in serum of cancer patients interacts with blood vascular endothelium and promotes secretion to the circulation of MCP-1, IL-6 and G-CSF. This leads to increased expression of adhesion molecules on the surface of endothelial cells that triggers endothelial-cancer cell interactions^18^.

The present work provides a deeper insight into the molecular basis underlying the mode of action of gal-8 and highlights its physiological relevance. We show that engagement of the LRP1 and uPAR receptors by gal-8 triggers a signaling cascade that involves activation of JNK and NFkB2, leading to increased SDF-1 and MCP-1 expression. Using a combination of inhibitors and siRNAs, we further show that uPAR signaling is not exclusively mediated by JNK, while the interactions between gal-8 and LRP1 is mediated through JNK.

Being an animal lectin, a large number of its biological activities depend upon the sugar-binding capacity of gal-8^9^. Accordingly, mutations of key residues of gal-8 involved in sugar binding, abrogate several of its biological activities, including promotion of cell adhesion and spreading^14^. Here we show that expression of MCP-1/SDF-1 and chemoattraction of cancer cells are induced by gal-8 mostly independent of its sugar-binding activity. They are not blocked by inclusion of a sugar inhibitor (TDG), while being induced by a gal-8 mutant (gal-8 W2Y) that lacks a sugar binding activity^14^. These results indicate that gal-8 binding to its cell-surface receptors (e.g. LRP1/uPAR) that triggers expression of MCP-1 and SDF-1 involves protein-protein, rather than protein-sugar interactions. In contrast, the effects of gal-8 on the expression of RANKL depend upon sugar-binding and involve interactions with MRC2^16^. Given that LRP1 is a negative regulator of RANKL expression^16^, while it is a positive regulator of SDF-1 expression, it is plausible that binding of gal-8 to LRP1, in a sugar-independent manner, negatively inhibits binding of gal-8 through its sugar-binding site, and therefore inhibits RANKL expression. The ability of gal-8, like other galectins, to engage in protein-protein interactions is well established^57–59^. Protein-protein interactions mediate gal-8 binding to NDP52, the autophagy cargo receptor^60^, while protein-protein interactions constitute part of the cytostatic effects of gal-1^61^.

The effects of gal-8 on cytokine/chemokine expression seem to have a physiological significance because total-body gal-8 KO mice^42^ show reduced expression of cytokines and chemokines, including TNF-α, RANKL, IL-1β, SDF-1, MCP-1, IL-6, and IP-10 while the opposite is true for gal-8 Tg mice^16^ that overexpress this lectin. The systemic reduction in cytokines and chemokines expression renders gal-8 KO animals partially resistant to growth and development of primary tumors and metastatic lesions. This is in accord with the notion that cytokines and chemokines promote growth of primary tumors, and support recruitment of cancer cells to the metastatic niche^33^. Hence, the action of gal-8 is most likely indirect and stems from its effects on the tumor microenvironment that secrets cytokines/chemokines in response to gal-8. This could account for the reduced size of the primary tumors, grown in gal-8 KO mice. This is also in accord with the observation that gal-8 does not control, in a cell-autonomous manner, primary growth of prostate cancer cells^28^.

Secreted MMP9 is a promoter of cancer metastasis. It acts by degrading the cellular matrix thus, supporting tumor cell invasion and spreading^62^. Here we show that gal-8 KO mice manifest reduced expression of MMP9 that could contribute to their resistance to tumor development. Gal-8 KO mice also have reduced expression of iNOS that can be activated by pro-inflammatory cytokines and may take part in anti-microbial activities^63^. While iNOS is commonly considered as being anti-tumorigenic, NOS2 has been implicated as a contributor to the process of tumor initiation and/or development, especially when the concentrations of its product, NO, are low. Therefore, inhibition of iNOS is proposed as a potential therapy in cases of triple-negative breast cancer^63^.

The third promoter of tumor growth whose expression is induced by gal-8 is Gas6, the ligand of the TAM family (Tyro3, Axl, and Mer) of receptor tyrosine kinases. Gas6 supports the development of several cancer types, (e.g. acute myelocytic leukemia, oral, prostate, renal, pancreatic, and ovarian cancers)^64^ and its expression is associated with poor prognosis^65^. The ability of gal-8 to promote expression of Gas6, and the observed reduced expression of Gas6 in gal-KO mice, points at gal-8 as a potential physiological inducer of Gas6 expression that supports tumor progression.

Additional mechanisms may contribute to the pro-metastatic action of gal-8. These include promotion of homotypic aggregation of the tumor cells as well as increased cell-matrix interactions that increase cell growth, adhesion, and selective metastatic seeding^28,30,31^. This can be attributed to the role of gal-8 as an extracellular matrix protein, equipotent to fibronectin in promoting cell adhesion, spreading and migration^20^. Accordingly, gal-8 silencing inhibits filopodia formation, and aggregation of cancer cells^28^; processes that are actively engaged in metastatic progression.

**Figure 6:**
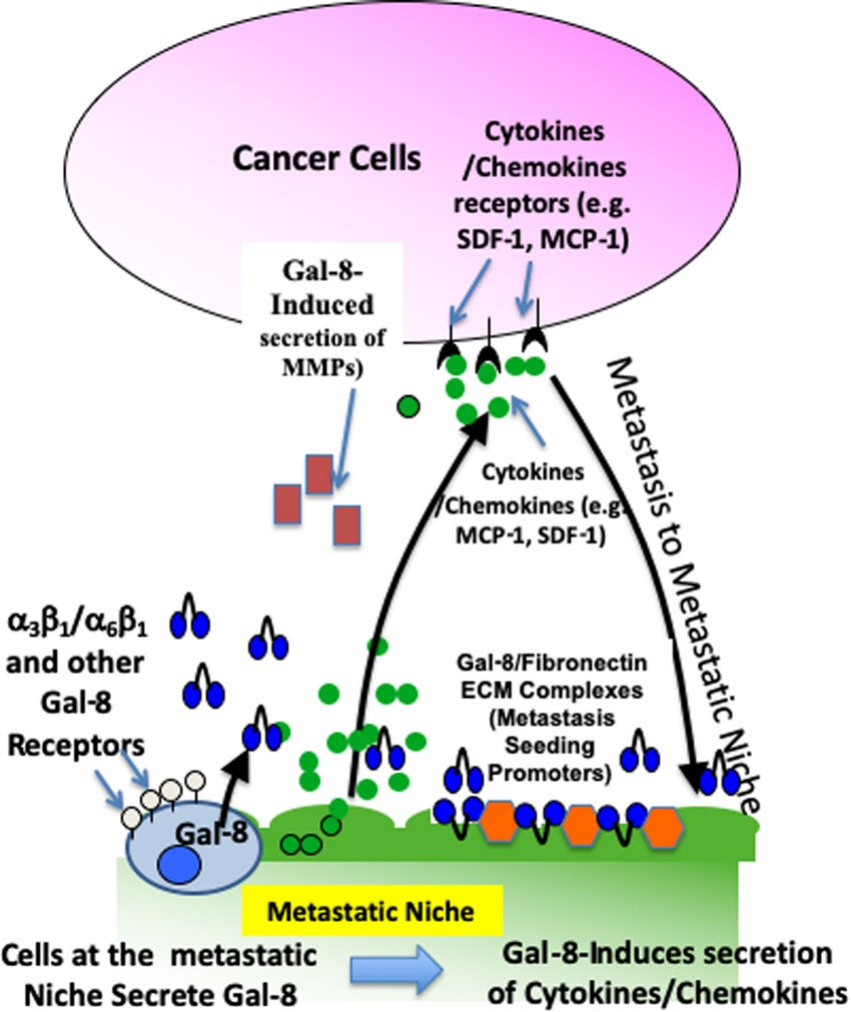
The ‘vicious cycle’ induced by Gal-8 to promote tumor growth and metastasis. Gal-8 drives cancer growth and metastasis by at least two mechanisms: i. Promotion of cell-matrix interactions that increase selective metastatic seeding through Gal-8 binding to α_3_β_1_ and α_6_β_1_ integrins, expressed by metastatic cells, that bind to Gal-8/fibronectin complexes at the metastatic niche and induction of MMPs; ii. Dissemination of gal-8, expressed by the primary tumor cells and by the tumor microenvironment that induces in an autocrine and paracrine manner the expression and secretion of cytokines and chemokines at the primary tumor site that promotes primary tumor growth. In addition, gal-8 secreted at the metastatic niche further enhances the production of cytokines/chemokines that chemoattract cancer cells to this site.

In summary, this study points at the existence of a ‘vicious cycle’ (Fig. 6) whereby gal-8 secreted both by tumor and naïve cells present in the tumor microenvironment, promotes in an autocrine and paracrine manner the secretion of chemokines, cytokines, and additional proteins (e.g. MMP9, GAS6) that support tumor growth and induce recruitment of cancer cells to the metastatic niche. The recruited tumor cells that secrete gal-8, further propagate this ‘vicious cycle’. Therefore, this study points at gal-8 inhibitors^66^ as potential new drugs in the combat of cancer and its adverse outcomes.

## Methods

### General

All methods were performed in accordance with the relevant guidelines and regulations in effect at the Weizmann Institute of Science, Rehovot, Israel, and were approved by the institute.

### Materials

Commercially available reagents were purchased from the following resources: Trypsin-EDTA, RPMI 1640 medium solution, Penicillin and L-Glutamine were from Biological Industries (Beit Haemek, Israel). Fetal bovine serum (FBS) was from Hyclone Laboratories Inc. (Logan, UT). Isopropyl-β-D-thiogalactopyranoside (IPTG) and PCR Master Mix (Dreamtaq) were purchased from MBI Fermentas (Amherst, NY). Dispase II (neutral protease, grade II) was from Roche Diagnostics (Mannheim, Germany). siRNA SMARTpool libraries were provided by Dharmacon (Lafayette, CO, USA). GST-coupled resins were from Navagen (Madison, WI). Thiodigalactoside (TDG) and CCR2 antagonist were from Santa Cruz biotechnology (Dallas, TX). Lipofectamine 2000 was from GIBCO-BRL (Grand Island, NY). PerfectPure RNA Cell & Tissue for RNA extraction was from 5PRIME (Hamburg, Germany). cDNA reverse transcription kit was purchased from Applied Biosystems (Carlsbad, CA). Real time PCR kit (SYBR green PCR master mix) was from Invitrogen (Carlsbad, CA). Protease inhibitor cocktail, Lactosyl-Sepharose beads, Wortmannin, lysozyme, Collagenase type 1 A, Dulbecco’s modified Eagle’s medium (DMEM), Diethyl pyrocarbonate (DEPC) and AMD3100 were from Sigma Chemicals Co. (St. Louis, MO). “Ready-To-Glow” secreted luciferase reporter system was from Clontech (Mountain View, CA, USA). Recombinant human SDF-1 was from R&D systems Inc. (Minneapolis, MN). Gal-8 was a bacterially expressed recombinant protein, encoded by the cDNA of rat gal-8^13^. GST-gal-8 and the GST-Gal-8-W2Y mutant were generated as described^14^.

### Antibodies

Polyclonal anti-SDF-1, monoclonal antibodies against phospho-JNK (Y185/Y223), JNK, and  IKK α + β, and polyclonal phospho-IKK α + β (S180/S181) antibodies were from Abcam PLC (Cambridge, UK). Polyclonal actin and polyclonal Akt antibodies were from Santa Cruz Biotechnology (Dallas, TX). Monoclonal phospho-Akt (Ser 473) and polyclonal IkB-α antibodies were from Cell Signaling Technology, Inc. (Beverly, MA). Monoclonal anti-vinculin antibodies were kindly provided by the lab of Prof. Geiger (Weizmann Institute of Science, Rehovot, Israel). Peroxidase-conjugated affinity purified goat anti-mouse, goat anti-rabbit and donkey anti-goat antibodies were all from Jackson ImmunoResearch Laboratories (West Grove, PA).

### Agglutination activity of gal-8

Hemagglutination activity was measured by mixing serial dilutions of gal-8 in PBS (50 μL/ well) with packed rabbit erythrocytes in PBS (50 μL/ well) in micro-titer U-shape plates. Following 1 h incubation at 22 °C, the agglutination activity was determined as described^19^.

### Animals

Gal-8 KO mice and their wild-type controls (*denoted WT1*), as well as Gal-8 Tg mice and their wild-type controls (*denoted WT2*) were generated as we previously described^16,42^. Gal-8 KO mice underwent 7 backcrosses to C57Bl/6 J mice. The WT2 animals were CB6F1 mice as described^16^. All animals were housed under standard light/dark conditions in the animal care unit of the Weizmann Institute of Science. Mice were given food and water *ad libitum*. Experiments were approved by the Animal Care and Use Committee of the Weizmann Institute of Science.

### Cell lines

E0771 breast cancer cells derived from C57BL mice were purchased from CH3 BioSystems (Amherst, NY). D122-Luc murine lung cancer cells expressing luciferase, were kindly provided by Prof. Lea Eisenach (Weizmann Institute of Science). Cells were grown according to the ATCC instructions. Primary osteoblasts from calvariae of newborn mice and long bones of adult mice were isolated as we described^16^.

### Primary tumor models

5 ×10^5^ E0771 cells were injected into the 4^th^ mammary gland of 9-weeks old C57BL/6 J female mice. Mice were sacrificed 6 weeks post injection and the developed tumors were weighted and photographed.

### Lung Metastasis models

5 ×10^5^ D122 cells were injected into the tail-vein of C57BL/6 J male mice. Six weeks post injection mice were sacrificed and the lungs were extracted, weighted, and examined for the presence of cancerous lesion.

### RNA analysis

Cells were grown in 6- or 12-well plates. Following treatment, cells were harvested and total RNA was extracted using the PerfectPure RNA kit (5-prime). RNA was quantified and cDNA was generated by cDNA Reverse Transcription kit (Applied Biosystems) following the manufacturer instructions. Quantitative detection of mRNA transcripts was carried out by real-time PCR using ABI-Prism 7300 instrument (Applied Biosystems) using SYBR Green PCR mix (Invitrogene) and specific primers (400 nM final concentration) (Supplemental Table S1). Results were normalized to mRNA levels of β-actin or HPRT. For Microarray analysis, RNA was extracted as above and underwent hybridization to mouse GeneST 2.0 microarrays (Affymetrix, Santa Clara, CA). Results were analyzed as described^42^. The microarray data have been deposited in NCBI’s Gene Expression Omnibus^67^ and are accessible through GEO Series accession number GSE106631 (https://www.ncbi.nlm.nih.gov/geo/query/acc.cgi?acc =GSE106631).

### RNA extraction from murine long bones

Femur and tibia were removed and placed in RNAlater solution (Ambion, Foster City, CA). The bone marrow was flushed out, and the remaining bones were cut into 1–2 mm^3^ pieces and crushed. Total RNA was extracted and analyzed as detailed above.

### RNA extraction from murine liver, kidney and lung

Nine weeks old CD1 mice or 7 week old WT1 and gal-8 KO mice were sacrified and liver, kidneys and lungs were removed. Single cell suspensions were made and were plated in growing medium for 24 h at 37 ^o^C. Cells were then treated with 50 nM gal-8 or SFM (control) for 24 h. Cells were harvested, total mRNA was extracted and qRT-PCR was conducted as described above. HPRT served as a control for normalization purposes.

### siRNA transfections

Osteoblasts were transfected with siRNA SMARTpool (Dharmacon), using Lipofectamine 2000 transfection reagent (GIBCO-BRL, Grand Island, NY) according to manufacturer’s instructions. Briefly, 50 pmol (2.5 μl of 20 μM stock) of siRNA solutions were diluted in 50 µl of serum-free medium. 1 μl Lipofectamine 2000 was diluted in the same volume of serum free medium. Both solutions were mixed together and incubated at room temperature for 15 minutes. The mix was added 1:10 to the osteoblasts’ culture medium. Incubation with the siRNA solutions was carried out for 48 h.

### Western blot analysis

Cells were harvested in lysis buffer (25 mM Tris/HCl, 25 mM NaCl, 0.5 mM Ethylene Glycol Tetraacetic Acid (EGTA), 2 mM sodium orthovanadate, 10 mM NaF, 10 mM sodium pyrophosphate, 80 mM β-glycerophosphate, 1% Triton X-100, 0.05% Sodium Dodecyl Sulfate (SDS) and protease inhibitors 1:1000, pH 7.5) and were centrifuged at 12,000 × *g* for 20 min at 4 °C. Supernatants were collected, and samples of 50 μg protein were mixed with 5 × Laemmli sample buffer and were resolved by SDS-PAGE under reducing conditions. Proteins were transferred to nitrocellulose membranes for Western blotting with the indicated antibodies.

### Wound healing assay

Wound-healing assays were performed according to manufacturer instructions. In brief, ibidi culture-inserts were placed in 24-well plates. Osteoblasts were seeded in one of the insert chambers (~70,000 cells) and incubated at 37 ^o^C for 24 h. The osteoblasts medium was replaced with serum-free medium with or without 50 nM gal-8, and PC3 cells were seeded in the second insert chamber (~35,000). The cells were further incubated at 37 ^o^C for 24 h. The culture medium was then replaced with fresh serum-free medium, the culture-inserts were removed (time zero), and the cells were further incubated at 37 ^o^C for 6 h. The gap between the inserts was photographed using IX2-UCB Olympus camera at time 0 and 6 h.

### Quantification of soluble SDF-1 and MCP-1

Media from cells were used for quantification of secreted SDF-1 and MCP-1 using a murine SDF-1 and MCP-1 ELISA Development Kit (PeproTech, Rocky Hill NJ) according to the manufacturer instructions. SDF-1 and MCP-1 amounts were normalized to total cellular protein concentration, and were quantified by Bradford assay.

### pMET-Luciferase assay

Osteoblasts were transfected with pNFκB-MetLuc2-Reporter, pMET-Luc-SDF-1 or with a control p-MET-Luc2 vector and were treated with gal-8 24 h post transfection. NFkB or SDF-1 promoter activity in response to gal-8 was determined using the “Ready-To-Glow secreted luciferase Reporter System” according to the manufacturer’s instructions (Clontech, Mountain View, CA, USA). Secreted luciferase levels were measured using the TECAN infiniteM200 luminometer.

### Tumor volume analysis

Mice were scanned 3 weeks post injection of tumor cells to the mammary glands, using small animal *in-vivo* μCT scanner (TomoScope 30 S duo, VAMP, Germany) following instrument-operating instructions. Scans were performed using the 65-65-360-90 protocol (using two micro-focus x-ray tubes of 65 Kv with an integration time of 90 ms), with a resolution of 80 μm. Image reconstruction was carried out by the Impact View software (VAMP, Germany). Estimation of tumor volume based on *in-vivo* CT scans was performed using the Measure Stack volume measurement plugin in ImageJ (NIH).

### Statistics

Data are presented as mean ± SEM unless otherwise specified. Group means were compared using the non-paired t-test. Differences of p < 0.05 were considered significant.

## Supplementary information

Supplemental Material.
