# Supplementary material for: The Animal Lectin Galectin-8 Promotes Cytokine Expression and Metastatic Tumor Growth in Mice
Source: Sci Rep. 2020 Apr 30;10:7375. doi: 10.1038/s41598-020-64371-z (PMC7193594; doi:10.1038/s41598-020-64371-z)
Supplement: Supplementary file 1 — Supplemental Material. [file 41598_2020_64371_MOESM1_ESM.pdf]

The Animal Lectin Galectin-8 Promotes Cytokine Expression and Metastatic Tumor Growth in Mice

Hadas Shatz-Azoulay[1], Yaron Vinik[1], Roi Isaac[1], Ulrike Koehler[1], Sima Lev[1], and Yehiel Zick[1*]

Figures S1-S13 , Table S1, and Figure legends

Figure S1, Shatz-Azoulay et.al.

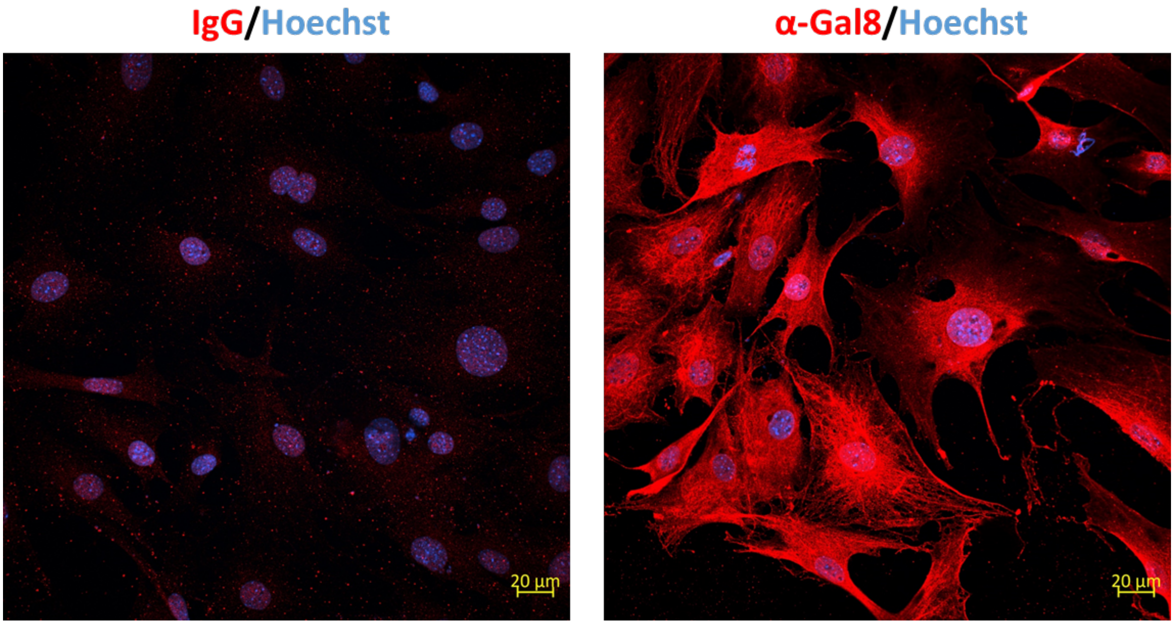

Figure S2, Shatz-Azoulay et.al.

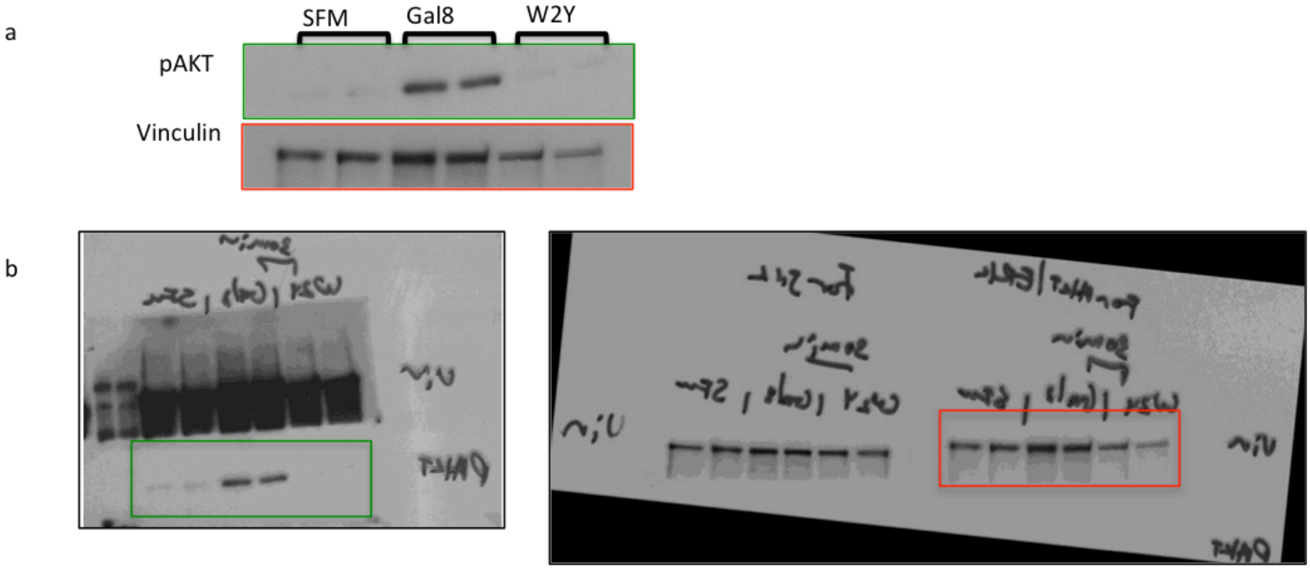

Figure S3, Shatz-Azoulay et.al.

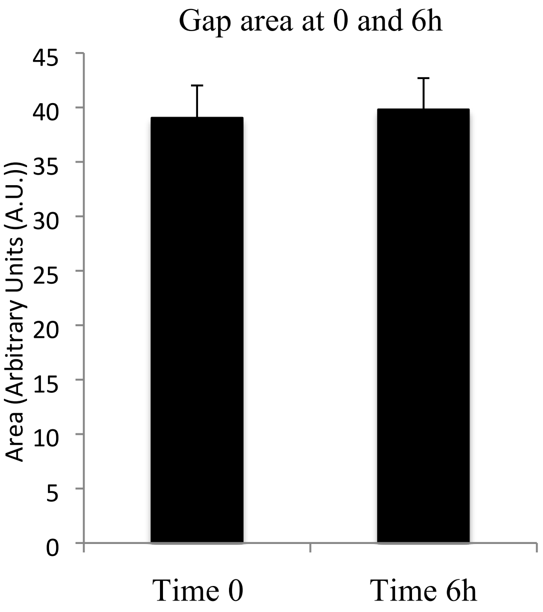

Figure S4, Shatz-Azoulay et.al.

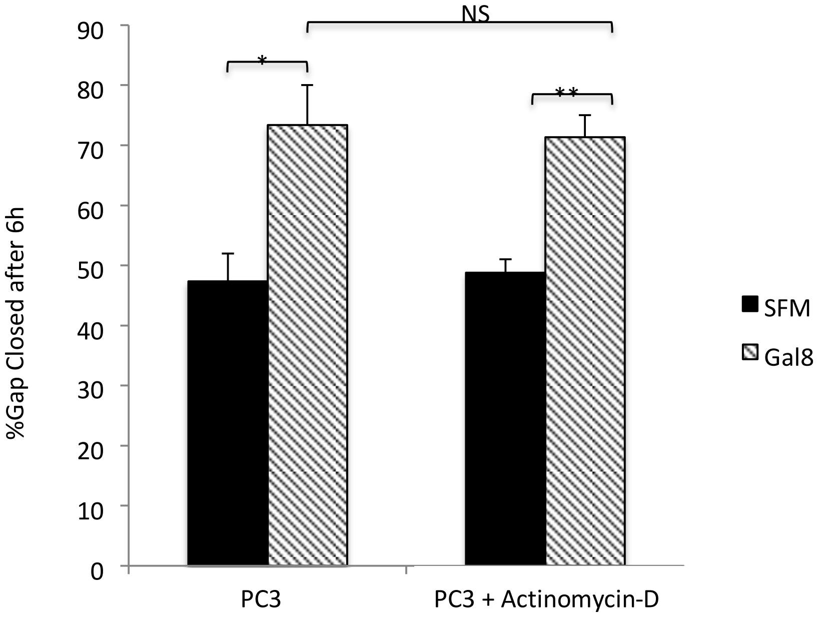

Figure S5, Shatz-Azoulay et.al

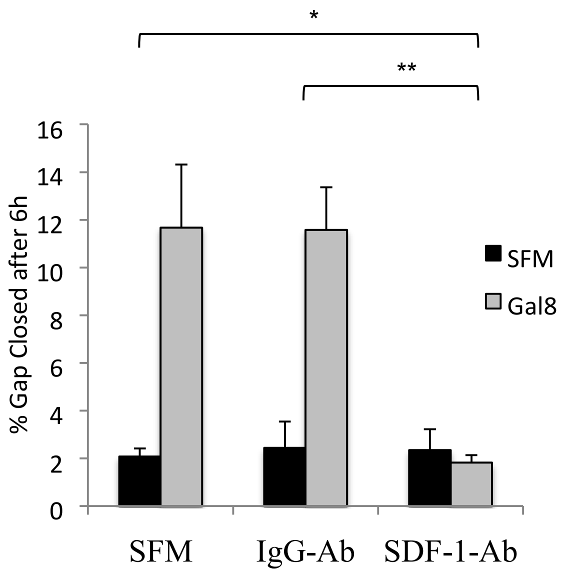

Figure S6, Shatz-Azoulay et.al

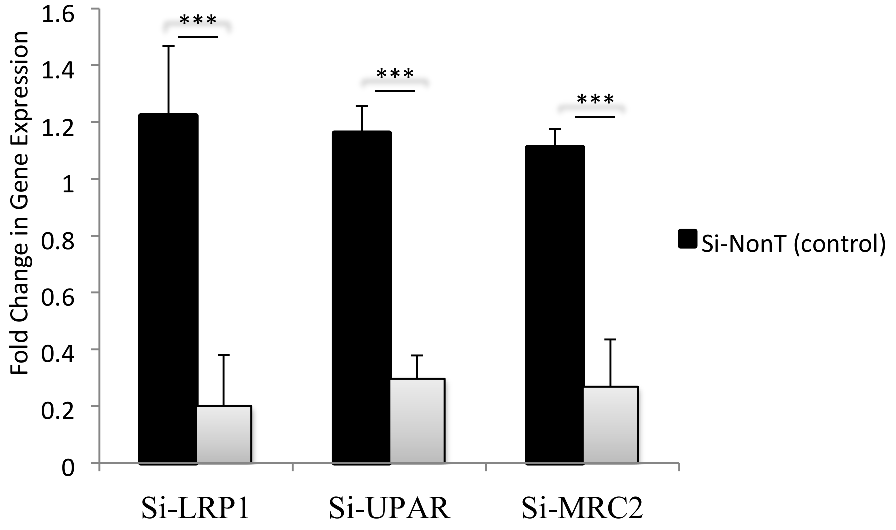

Figure S7, Shatz-Azoulay et.al

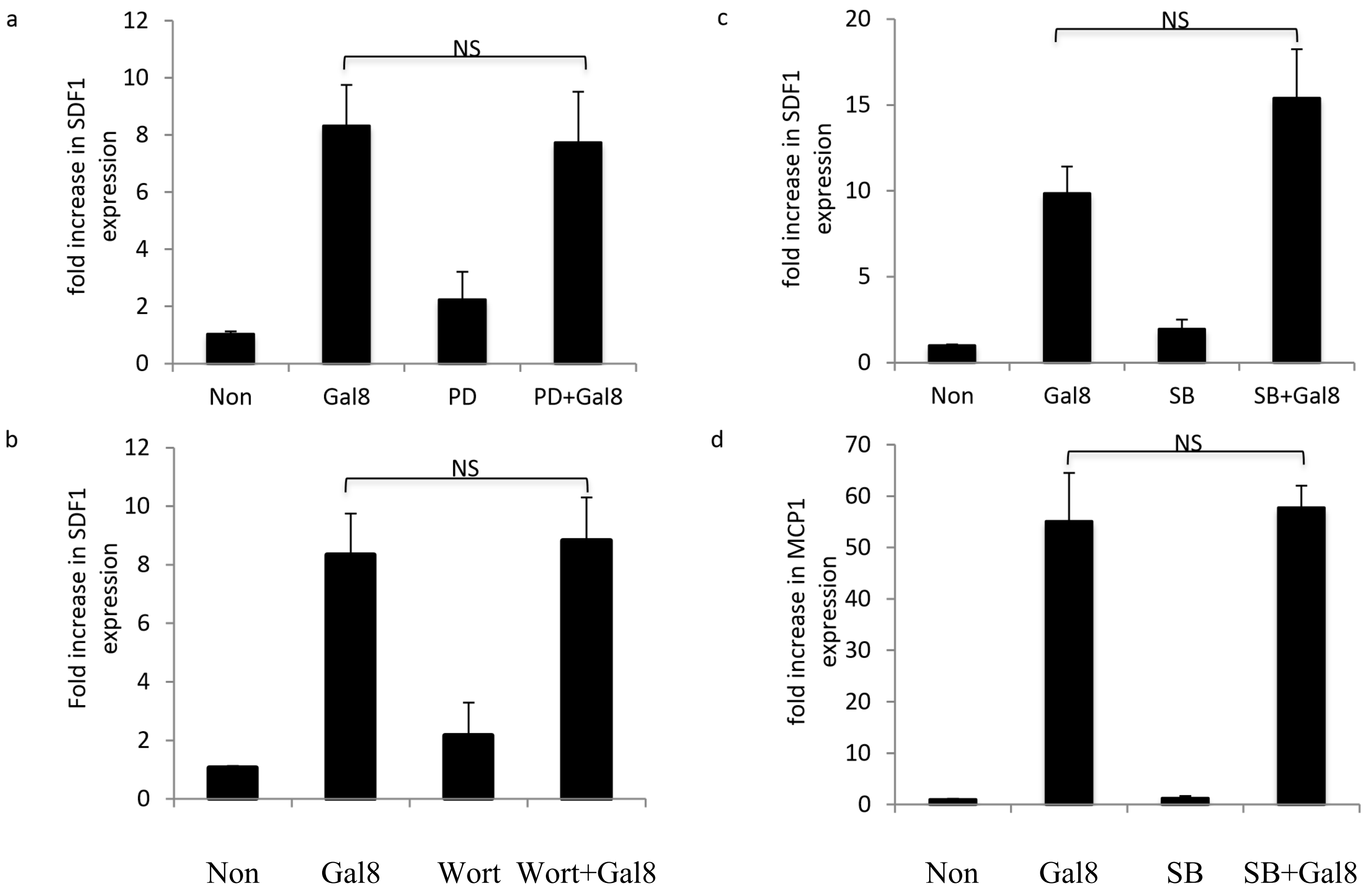

Figure S8, Shatz-Azoulay et.al

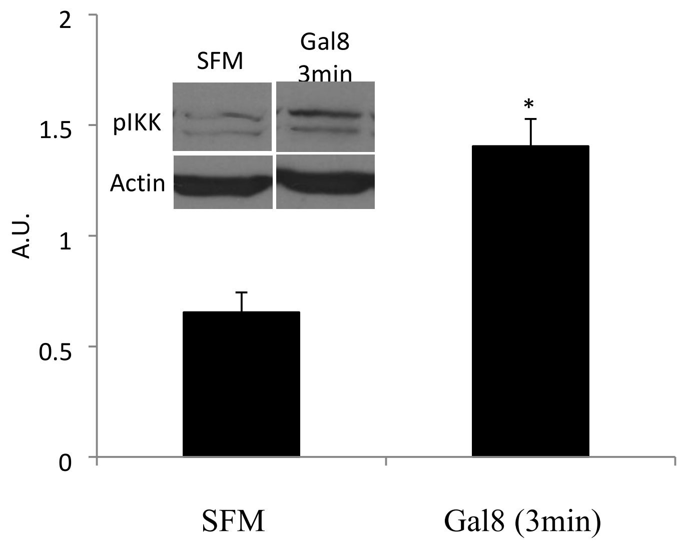

Figure S9, Shatz-Azoulay et.al

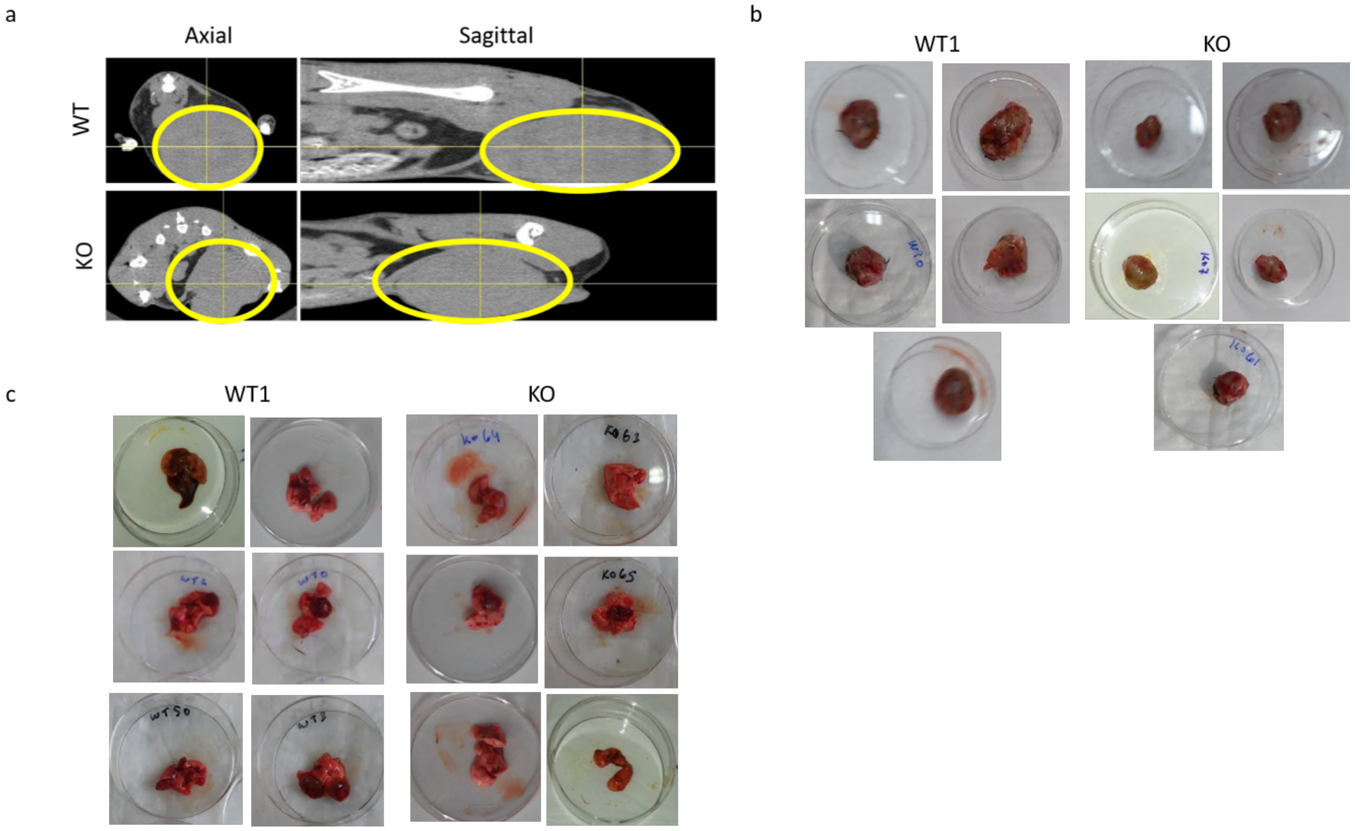

Figure S10, Shatz-Azoulay et.al

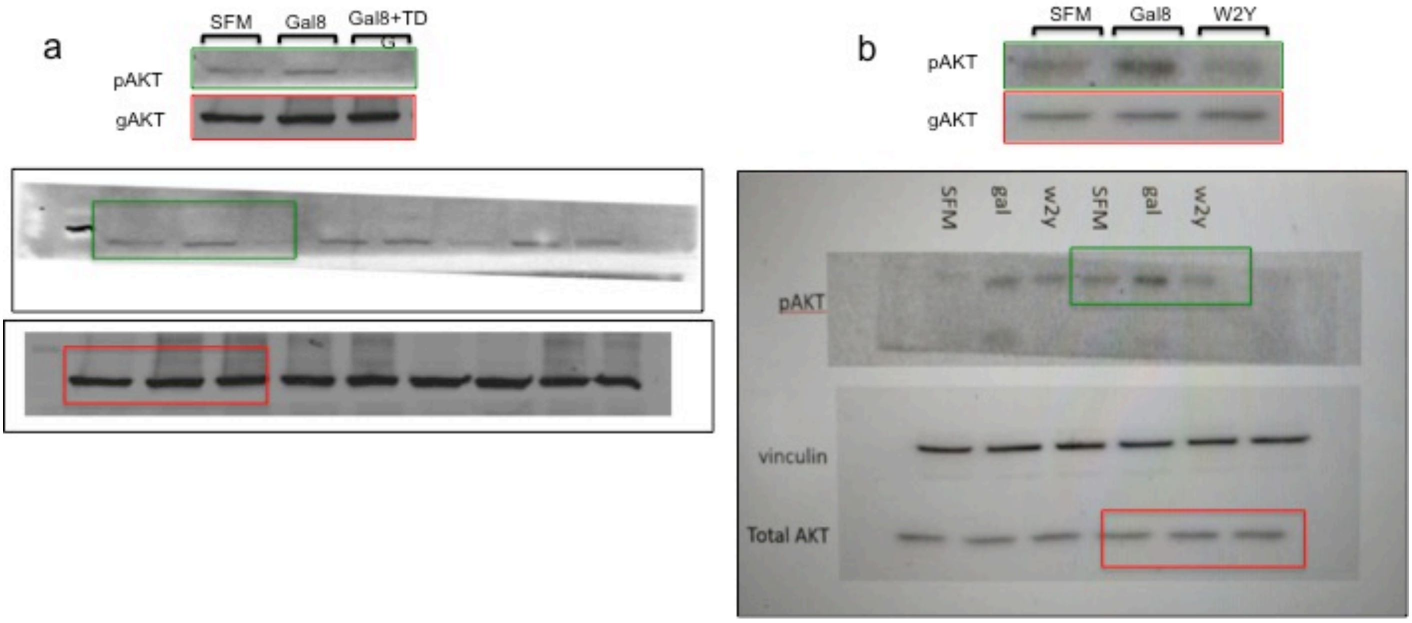

Figure S11, Shatz-Azoulay et.al

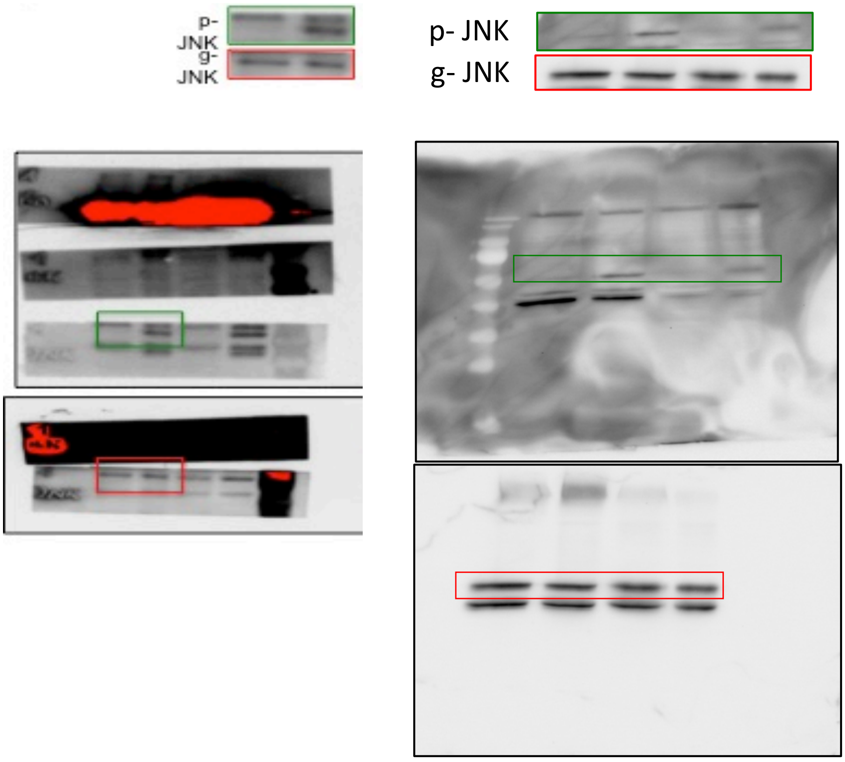

Figure S12, Shatz-Azoulay et.al

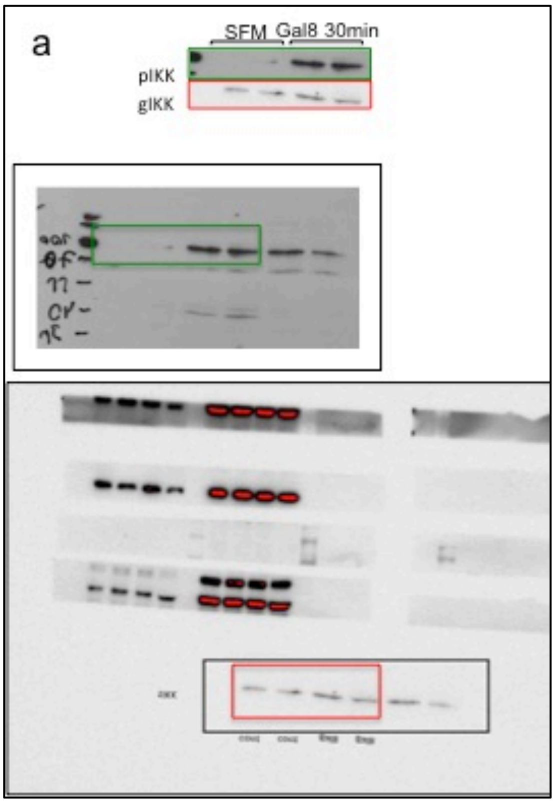

Figure S13, Shatz-Azoulay et.al

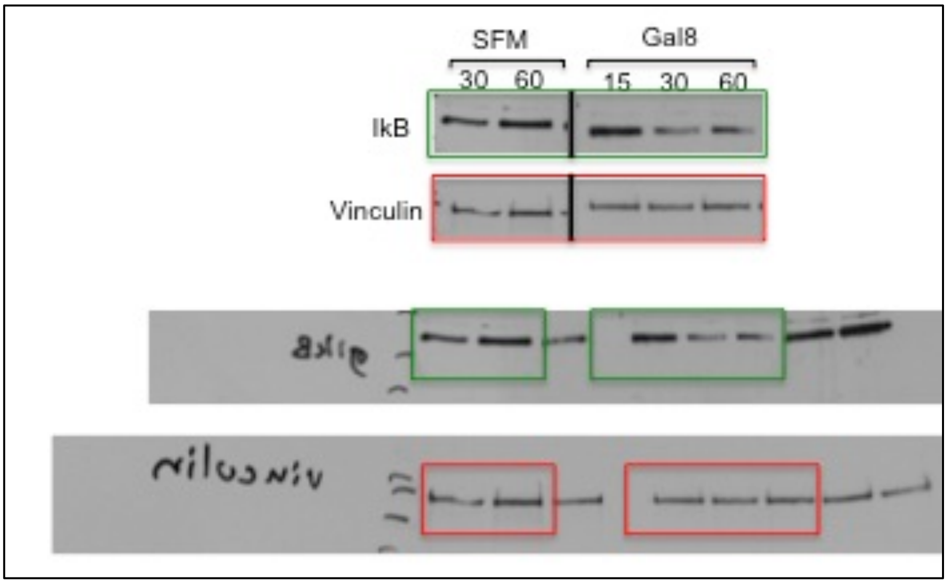

Table S1: qRT-PCR primer sequences (5′–3′)

Gene	Forward primer	Reverse primer
RANKL	ATCGGGAAGCGTACCTACAG	GTGCTCCCTCCTTTCATCAG
SDF-1	CGTGAGGCCAGGGAAGAGT	GATGAGCATGGTGGGTTGA
MCP-1	AGGTGTCCCAAAGAAGCTGTA	A TGTCTGGACCCA TTCCTTCT
IP-10	TCCTTGTCCTCCCTAGCTCA	ATAACCCCTTGGGAAGATGG
iNOS	GCCCTGCTTTGTGCGAAGTG	AGCCCTTTGTGCTGGGAGTC
IL-6	ATGGATGCTACCAAACTGGAT	TGAAGGACTCTGGCTTTGTCT
IL1-β	TCAGGCAGGCAGTATCACTC	ACGGGAAAGACACAGGTAGC
TNF-α	CCAGTGTGGGAAGCTGTCTT	AAGCAAAAGAGGAGGCAACA
MMP9	GCCACCACAGCCAACTATGA	AGACGCACAGCTCTCCTGC
MRC2	GCCATACGGCTTTGCCCTAC	GGCCCTGGATTCGGAAACAC
uPAR	TGTGCTGGGAAACCGGAGTT	GAGGTGGGTCGGGAAGGAGT
LRP1	TCAGACGAGCCTCCAGACTCT	ACAGATGAAGGCAGGGTTGGT

**Gene**	**Forward primer**	**Reverse primer**
Gal-8 (mouse)	TGAACACCAATGCCCGAAGC	GCGTGGGTTCAAGTGCAGAG
Gal-8 (rat)	TGTATGCCCACAGGATCAAC	ATCCGAGCTGAATCTGAACC
Gas-6	ATGAAGATCGCGGTAGCTGG	CCAACTCCTCATGCACCCAT
β-Actin	GGCCAACCGTGAAAAGATGA	CACAGCCTGGATGGCTACGT
HPRT	GCAGTACAGCCCCAAAATGG	GGTCCTTTTCACCAGCAAGCT

Legends to Supplemental Figures

Fig S1– Immunofluorescence staining of gal-8 in osteoblasts. Osteoblasts from newborn CD1 calvaria were grown on cover slips overnight, fixed with 4% PFA for 20 minutes, and blocked in blocking solution (10mM Tris pH 7.5, 150mM NaCl, 1% glycine, 2% BSA, 10% goat serum, 0.1% triton) for 1 hour. The slides were then incubated with 1st antibodies (monoclonal 106.1 anti-gal 8 antibodies (1:100) [1], or control IgG for 1 hour, and then with 2nd antibodies (anti-mouse cye3, 1:400) for 1 hour. Hoechst was used to stain the nucleus. Fluorescence images were acquired using laser confocal microscope 606 (LSM800, Zeiss).

Fig S2. Effects of gal-8 on Akt activity. Primary osteoblasts were treated with GST-gal-8 or with GST-W2YGal8 (50nM) for 30min. Cells were harvested, total protein was extracted and was analyzed by Western blotting using antibodies specific for the phosphorylated forms of AKT (pAKT) or Vinculin for loading control. A representative experiment is shown (**a**). The full-length blots are shown (**b**).

Fig. S3- Osteoblasts fail to migrate within 6h of treatment. Ibidi culture-Inserts were placed in 24 well plates, and osteoblasts were seeded in both sides of the insert chambers (70,000 cells) and incubated at 37°C. 24 hours later the osteoblasts medium was replaced with serum-free medium with galectin-8 (50nM). 24 hours thereafter, the Culture-inserts were removed with sterile tweezers and 1ml of serum-free medium was added to the wells. The cells were incubated at 37°C for 6 h, and the gap created by the inserts was photographed using Nikon Digital sight DS-U3 camera at time 0, and 6 h. The area between the original marked gap lines and the position of the front-line of the cultured osteoblasts was measured. n=6 independent experiments carried out in duplicates.

Fig. S4 Actinomycin-D does not affect PC3 cell migration towards gal-8-treated osteoblasts. Ibidi Culture-Inserts were placed in 24-well plates, and osteoblasts were seeded in one of the insert chambers (70,000 cells) and incubated at 37°C. 24 hours later the osteoblasts medium was replaced with serum-free medium with or without gal-8 (50nM) and PC3 cells were seeded in the insert's second chamber (35,000 cells). 24 hours thereafter, the culture-inserts were removed with sterile tweezers and 1ml of serum-free medium with or without Actinomycin-D (2.5 μg/ml) was added to the wells, as indicated. The cells were incubated at 37°C for 6 h, and the gap created by the inserts was photographed using Nikon Digital sight DS-U3 camera at time 0, and 6 h. (n=3 independent experiments carried out in duplicates ***p<0.001). NS= non significant.

Fig S5. Anti SDF-1 antibodies selectively inhibit Gal-8-induced gap closure between osteoblasts and PC3 Cells. Osteoblasts (70,000 cells) were seeded in one chamber of 'Ibidi' Culture-Inserts and were incubated at 37°C. 24h later the osteoblasts were treated with gal-8 (50nM) or SFM (control) while PC3 cells (35,000 cells) were seeded in the second chamber. 24h thereafter the culture-media was replaced with gal-8-free medium. SDF-1 antibody (2µg/ml) or control IgG were added to the culture medium and the 'Ibidi' inserts were removed. The cells were further incubated at 37°C for 6h. The gap between the two cultures was photographed and quantified at the time of removal of the 'Ibidi' insert (time 0) and 6 h later. Results shown are means±SEM of triplicate measurements (*p<0.05; **p<0.01; vs. untreated controls).

Fig S6. SiRNAs silence LRP1 UPAR and MRC2 gene expression. Osteoblasts (5x10 cells) were transfected with siRNAs directed against LRP1, uPAR, MRC2 or control non-targeting siRNA. 72 h thereafter gal-8 (50nM) was added for another 24 h, after which the cells were harvested, total mRNA was extracted and qRT-PCR was conducted for LRP1, uPAR, and MRC2 mRNA. HPRT served as a control for normalization purposes. Results shown are means±SEM of 3 experiments done in duplicates. (***P<0.001 vs. untreated controls).

Fig. S7- Inhibitors of ERK, Akt and p38 do not affect gal-8-induce: SDF-1/MCP-1 expression. Osteoblasts extracted from calvariae of newborn mice were seeded in 12-well plates ($5x10^4$ cells per well). 24 h later the medium was replaced with serum-free medium with or without inhibitors of MEK (PD98095 25mM) (**a**), AKT (Wortmannin 1mM) (**b**) or p38 (SB203580 15mM) (**c-d**). 2 h later gal-8 (50nM) was added for another 24 h, after which the cells were harvested, total mRNA was extracted and qRT-PCR was conducted in order to quantify changes in SDF-1 (**a-c**) and MCP-1 (**d**). HPRT served as a control for normalization purposes. Results shown are mean±SD of 4 experiments done in duplicates. (*p-value<0.05, **p-value<0.01). NS= non-significant.

Fig. S8- Short (3 min) treatment of osteoblasts with gal-8 induces phosphorylation of IKKα/β. Osteoblasts extracted from calvariae of newborn mice were seeded in 6-well plates (10^5 cells /well). 24 hours later cells were treated with Gal-8 (50nM) for 3 min, after which the cells were harvested, total protein was extracted and analyzed by Western blotting using antibodies specific for the phosphorylated forms of IKK α/β. Data shown is a representative of 2 experiments done in duplicates. (*p-<0.05)

Fig. S9. Gal-8 KO mice manifest reduced primary tumor growth and reduced metastatic potential *in vivo*. (**a,b**) $5x10^5$ E0771 cells were injected subcutaneously into the 4th mammary gland of wild type (WT1) and gal-8 KO female mice (9 weeks old). 3 weeks post injection the mice were scanned by i*n-vivo* μCT (**a**), sacrificed, and the tumors developed in their mammary glands were photographed (**b**). $5x10^5$ D122-Luc cells were injected into the tail vein of WT1 and gal-8 KO male mice (9 weeks old). 6 weeks post injection mice were sacrificed and their lungs were photographed (**c**).

Fig. S10. Full-length blot of Fig. 1i (a) and 1j (b)

Fig. S11. Full-length blots of figures 3d

Fig.S12. full-length blots of figures 3h

Fig. S13. Full-length blots of figures 3j

References for Supplemental Material

1 Levy, Y. *et al.* Galectin-8 functions as a matricellular modulator of cell adhesion. *J Biol Chem* **276**, 31285-31295 (2001).
